# Structural adaptability of SARS-CoV-2 Nsp1 with the host network

**DOI:** 10.1007/s00249-025-01762-y

**Published:** 2025-06-14

**Authors:** Monikaben Padariya, Ted Hupp, Umesh Kalathiya

**Affiliations:** 1 International Centre for Cancer Vaccine Science, University of Gdansk, Ul. Kładki 24, 80-822 Gdansk, Poland; 2 Institute of Genetics and Molecular Medicine, University of Edinburgh, Edinburgh, EH4 2XR Scotland, UK

**Keywords:** SARS-CoV-2, Nsp1, Leader protein, 40S ribosome, Protein–protein interactions, Cyclophilins

## Abstract

The SARS-CoV-2 non-structural protein 1 (Nsp1) acts at multiple points toward the host cell to trigger its mRNA cleavage and decay. Nsp1 is found binding with the 40S ribosomal subunit and inhibiting the translation process, as well as docking with different cyclophilins. Herein, we evaluated the structural physicochemical properties of SARS-CoV-2 Nsp1 protein implementing different computational techniques. The Nsp1 was found to form a structured α-helical C-terminal region, following a conformational switch at residue S166 that is necessary for binding the 40S ribosome subunit. Similarly, the presence of cyclophilins stabilizes the Nsp1 C-terminus making a tilt movement at position 166. In the 40S ribosome-Nsp1 machinery, both the ribosomal uS3 and eS30 components were found equally interacting with Nsp1, which guided construction of their pharmacophores. Among a set of studied cyclophilins, FKBP1B showed the highest affinity with Nsp1 and PPIH made least interactions. The majority of cyclophilins dock to the conserved Nsp1 loop or linker region, which connects the C-terminus to the central domain. Our findings revealed that Nsp1 has a versatile C-terminus region which changes its conformations with respect to its host binding partner. Identified novel binding sites within the Nsp1 can assist in understanding its networking (in current or future such infections), as well as support drug discovery programs aimed at targeting the coronavirus family.

**Supplementary Information:**

The online version contains supplementary material available at 10.1007/s00249-025-01762-y.

## Introduction

**Fig. 1:**
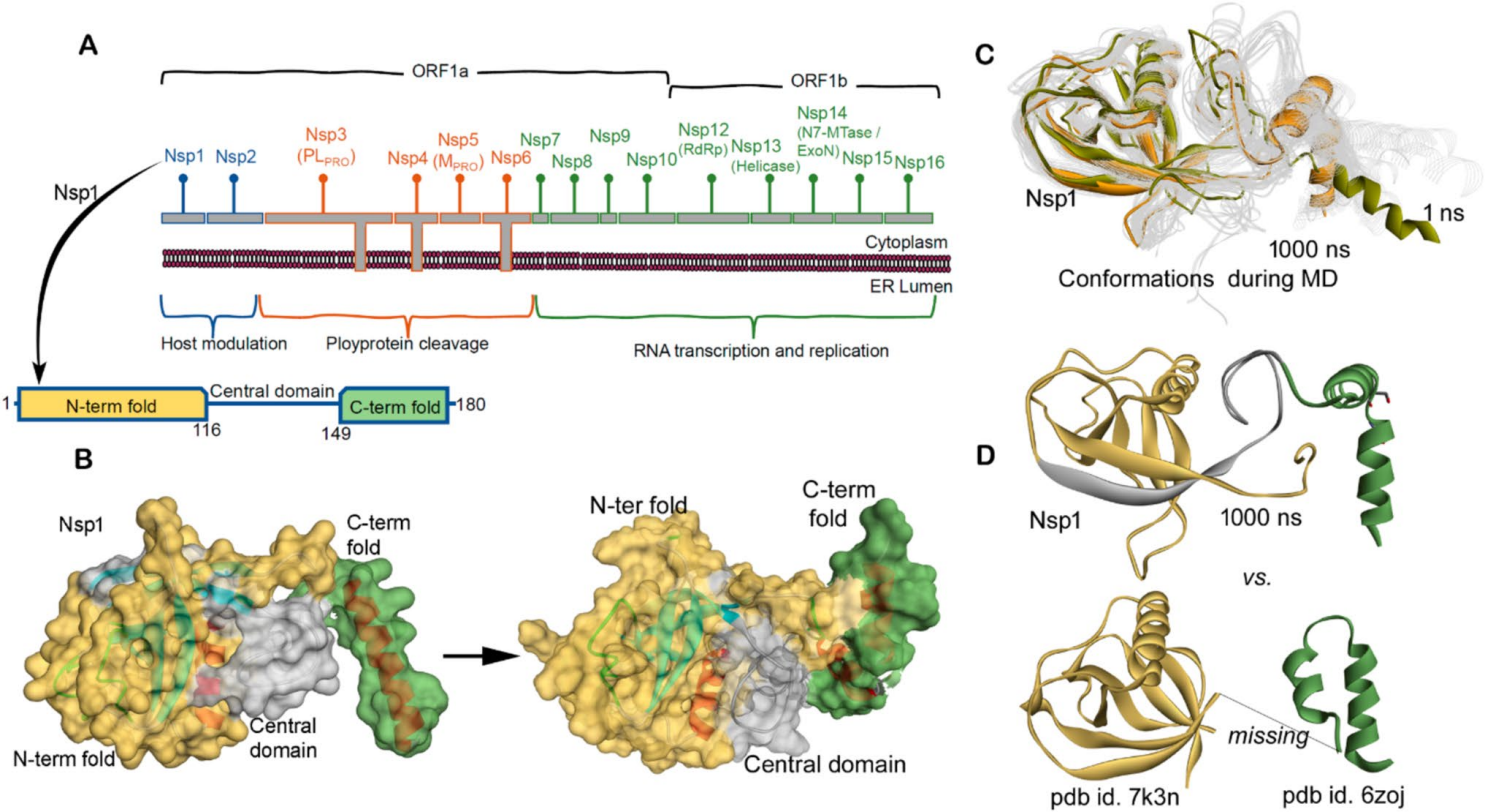
The SARS-CoV-2 non-structural leader protein (Nsp1). **A** The genomic structure of the virus codes for 16 non-structural proteins (Nsp1-16 merged as a polyprotein) (Sahin 2020). The bottom diagram presents different domains of Nsp1 protein (Mendez et al. 2021). **B** Protein models of SARS-CoV-2 Nsp1 (Mendez et al. 2021) built using I-TASSER (Roy et al. 2010) with highlighted domains in different colors. The right panel represents the conformational switch of the C-terminal domain after 1000 ns of molecular dynamic simulations (MDS). **C** Structural conformations observed during MD simulations for Nsp1 unbound state. **D** The Nsp1 structure retrieved from the end of the 1 µs simulation compared with the known cryo-EM structures (pdb id.: 7k3n (Semper et al. 2021) or 6zoj (Schubert et al. 2020)

The causative agent of the coronavirus disease 2019 (severe acute respiratory syndrome coronavirus 2; SARS-CoV-2) cause of the recent pandemic is a single-stranded RNA virus with envelope glycoproteins (Coronaviridae Study Group of the International Committee on Taxonomy of Viruses 2020; Sahin 2020). The SARS-CoV genome is composed of at least 14 functional open-reading frames (ORFs, Fig. 1A), leading to the expression of up to 29 structural and non-structural proteins (Perlman and Netland 2009). Besides the structural proteins and other accessory proteins (Fig. 1A), there is genomic structure of the virus codes for 16 non-structural proteins (NSPs; 1–16 merged as a polyprotein ORF1) (Sahin 2020; Perlman and Netland 2009). The Nsp1 protein referred to as the leader protein has been reported to have multiple functions toward the host (Fig. S1) (Pfefferle et al. 2011; Roy et al. 2010). Multifunctional Nsp1 protein triggers host cell mRNA cleavage and decay, as well as induces cytokines and chemokines [Kamitani et al. 2006; Baranvskiy et al. 2024; Kamitani et al. 2009; Huang et al. 2011; Law et al. 2007; Bujanic et al. 2022]. In addition, the SARS-CoV-2 Nsp1 protein binds to the 40S ribosomal subunit and inhibits translation (Figure S1) [Narayanan et al. 2015; Thoms et al. 2020] via inserting its C-terminal domain into the ribosomal mRNA channel and interfering with the mRNA binding [12-15]. Nsp1 degrades the antiviral immune response of the human host and serves as a virulence factor from coronaviruses which targets cellular processes to inhibit gene expression, downregulates type I interferon (IFN) response, and targets antiviral signaling pathways [Baranvskiy et al. 2024, Kamitani et al. 2009]. It has been reported that an alteration in the region of 160-173 amino acids (C-terminus; K164A and H165A mutants) of SARS-CoV Nsp1 led to the inactivation of the protein with ribosome [Baranvskiy et al. 2024]. Nsp1 is found to interfere with the nuclear export of cellular mRNAs (nucleocytoplasmic) via binding the host mRNA and hinders crucial networks of NXF1 to mRNA [Zhang et al. 2021; Vasudevan and Baraniuk 2021; Mei et al. 2024], as well as binding with the DNA polymerase α(Pol α)-primase [Kilkenny et al. 2022; Baranvskiy et al. 2024]. Genome-wide analysis [Bujanic et al. 2022] of protein-protein interactions (PPIs) between the SARS-CoV and human components have been carried out via a high-throughput yeast two hybrid screen [Bujanic et al. 2022; Coronaviridae Study Group of the International Committee on Taxonomy of Viruses 2020]. Redundant interactions between Nsp1 and a group of host proteins with peptidyl-prolyl cis-trans-isomerase activity have been reported, involving the cyclophilins or immunophilins (PPIA, PPIG, PPIH, FKBP1A, and FKBP1B; Figure S1) [Bujanic et al. 2022; Corbeil et al. 2012]. These molecules modulate the Calcineurin/NFAT (nuclear factor of activated T cells) pathway which has an important role in immune cell activation (Feske et al. 2001; Hogan et al. 2003). The NFAT family of transcription factors encodes four calcium-regulated proteins among which three (NFAT1/2/3) are expressed in various cell types involving T cells, B cells, mast cells, natural killer cells, and eosinophils (Feske et al. 2001; Hogan et al. 2003; Liu et al. 2006). Though the binding of Nsp1 and immunophilins (Pfefferle et al. 2011; Gamble et al. 1996; Davis et al. 2010; Reidt et al. 2003; Schultz and Clardy 1998; Deivanayagam et al. 2000) is known, the molecular level details of these protein networks remain elusive, and therefore, applying structural (computational) biology techniques, we explored their PPIs.

SARS-CoV Nsp1 protein can alter host RNA processing and degradation, and one of the mechanisms hypothesized is through altering the intermolecular network in the nuclear RNA exosome complex (Jonasson 2020). RNA exosome complex is primarily recognized as an essential factor in the processing of the stable RNA species produced by RNA polymerases I-III, for instance rRNAs, small nuclear RNAs (snRNAs), small nucleolar RNAs (snoRNAs), and tRNAs (Allmang et al. 1999; Kilchert et al. 2016). An essential function of the exosome is the removal of RNAs that result from the cryptic and eukaryotic cells. The exosome has an important role in RNA quality control (Kilchert et al. 2016; Wlotzka et al. 2011; Pefanis et al. 2015). The eukaryotic RNA exosome is an evolutionarily conserved ribonucleolytic complex consisting of six RNase proteins (Rrp41, Rrp42, Rrp43, Rrp45, Rep46, and Mtr3) and three RNA-binding proteins (Sahin 2020; Kilchert et al. 2016; Makino et al. 2013; Wasmuth et al. 2014). Based on similar structural orientation of Nsp1 and Rrp46 protein (Fig. S2), it can be speculated that Nsp1 binding Rrp45 could mimic Rrp46 within the exosome complex [Kamitani et al. 2006]. Altogether the Nsp1 protein can act at multiple levels and represents an attractive target within the coronavirus family [Ma et al. 2022; Züst et al. 2007], and therefore, considering crucial roles of Nsp1 in different biological systems we investigated change in its PPIs over the simulation time. The SARS-CoV-2 couples global inhibition of translation through Nsp1, along with efficient translation of the viral mRNA to allow expression of viral genes [Chen and Weng 2002]. Several studies have reported about the full-length Nsp1 structure (pdb id.: 8aou) [Wang et al. 2023; Mei et al. 2024; Mendez et al. 2021] describing the N- and C-termini of unbound-state are predominantly unstructured though absence of a cognate ligand, with a possibility of forming α-helical secondary structure at the C-terminus (Figure 1). These known structures demonstrated that the Nsp1 structure consists of intrinsically disordered regions (IDR) in the middle to C-terminal region, however, this is later appeared to form a α-helical elements when they bind to a macromolecular ligand (e.g., 40S ribosomal subunit). The C-terminus in the solution is suggested to fold on to the lobular N-terminal domain [Wang et al. 2023; Mendez et al. 2021]. In addition, it has been reported that a set of SARS-CoV Nsp1 amino acids (aa) within the range 1-12 (N-terminal) and 129-179 (C-terminal) are flexibly disordered [Meng et al. 2023; Narayanan et al. 2008; Narayanan et al. 2015]. Modeled full-length Nsp1 [Clark et al. 2021] and cryo-EM (cryo-electron microscopy) [Chen and Weng 2002] structures suggest that 150-180 aa form helical regions when binding to 40S ribosome factors. Overall, these findings highlights the significance of Nsp1 [Clark et al. 2021, Parrinello and Rahman 1981; Pefanis et al. 2015; Vankadari et al. 2020; Frolov et al. 2023], and in particular, the structure proposed by Sakabura et al. [Parrinello and Rahman 1981] is closely compatible with the our constructed Nsp1 model (Figure 1). Considering the fact that Nsp1 consists of IDRs in the middle to C-terminal regions we investigated the Nsp1 model structures consisting of distinct starting positions, and further evaluated them using the molecular dynamics simulations (MDS; Figure S2). Retrieving the energy-minimized SARS-CoV-2 Nsp1 structure (unbound form) after 100 ns of MD simulation, it was further screened against different cyclophilins (PPIA, PPIB, PPIH, PPIG, FKBP1A, and FKBP1B; Fig. S1B). Merging cryo-EM and biochemistry techniques (Schubert et al. 2020), it has been reported the SARS-CoV-2 Nsp1 docks with the 40S ribosomal subunit near the mRNA entry site. Among the 40S ribosome components, the uS5, uS3, and eS30 genes were presented interacting mostly with the Nsp1 C-terminal region (Schubert et al. 2020). However, the cryo-EM complex consists only of the C-terminus of Nsp1 protein, and therefore, considering our energy-minimized full-length SARS-CoV-2 Nsp1 structure, we investigated it with different binding partners (uS5, uS3, and eS30) retrived from the 40S subunit (Fig. 1C). However, we believe that Nsp1 should be investigated with the entire 40S subunit to investigate allosteric effects of other components that may lack any direct contact with Nsp1. Furthermore, PPIs of Nsp1 with Rrp45 (from the exosome complex) were evaluated, along with a designed set of linear peptide motifs that were screened against Nsp1 (Fig. S1D). These self-derived peptides from a human gene can be the basis to design peptide-based modulators against Nsp1, combined with combinatorial chemistry or click-chemistry (Brankiewicz et al. 2023). Identifying key interacting residues within different Nsp1-cyclophilins or 40S ribosome or RNA exosome systems, our findings revealed that the leader protein (Nsp1) has a versatile C-terminus region, which could change its conformations with respect to its binding network. In addition, our molecular dynamics systems suggest that S166-G168 aa controls the conformation necessary to dock Nsp1 with the 40S ribosome.

## Results and discussion

### Conformational switch of the Nsp1 C-termini

Visualizing the dynamics (root-mean-square fluctuation; RMSFs) and secondary structure (Ramachandran plots) changes of different Nsp1 models (Figs. S2, S3) over 100 ns of MD simulation suggests that model #2 has formed a comparatively well-defined α-helices and β-sheets (Fig. 1B). Individual Nsp1 residues evaluated by their RMSF highlighted that model #2 formed a stable structure (Figs. S2, S3), and therefore, it was further investigated (Figs. 1 and S3). Over a 1 µs MD simulation, the Nsp1 gained several conformations (Figs. 1C, S3A, and S3D; Video S1), and in particular, the C-terminal region formed a tilt conformation at S166 position (Figs. 1 and S3). The N-terminal and central domains resemble the crystal structure (pdb id.: 7k3n) (Semper et al. 2021; Clark et al. 2021), and the C-terminal has a conformation as presented in the cryo-EM structures (pdb id.: 6zoj (Schubert et al. 2020); Figs. S1D and S3).

It is known that isolated SARS-CoV-2 Nsp1 consists of intrinsically disordered regions (1–12 aa and 129–179 aa) (Wang et al. 2021; Agback et al. 2021; Almeida et al. 2007), which are found stable when complexed with some ligand or protein (40S ribosome) (Schubert et al. 2020; Semper et al. 2021). The initial Nsp1 structure has shown some structurally disordered regions in the C-terminus, which during MD simulation formed a well-defined α-helix gaining different conformational states (Figs. 1C and S4). Due to such tilt movements from the C-terminal (α-helical) domain (Fig. S3A), the Nsp1 structure initially gained high RMSDs (~ 11.50 Å) which stabilized by the end of the simulation (~ 8 Å; Fig. S3B). Besides such changes in dynamics of the Nsp1 protein, a hydrophilic surface demonstration showed significant fluctuations. Extracted protein coordinates from different time frames suggest that the C-terminus is highly hydrophilic and RMSF fluctuations reach up to ~ 10 Å (Fig. S3A and S3C). Additionally, the simulated Nsp1 has formed consistent, stable intramolecular interactions (Fig. S3B, S3C), and the energy-minimized structure was used to investigate with cyclophilins or the 40S ribosomal subunit or exosome components.

### Cyclophilins sharing a common interface with Nsp1

**Fig. 2:**
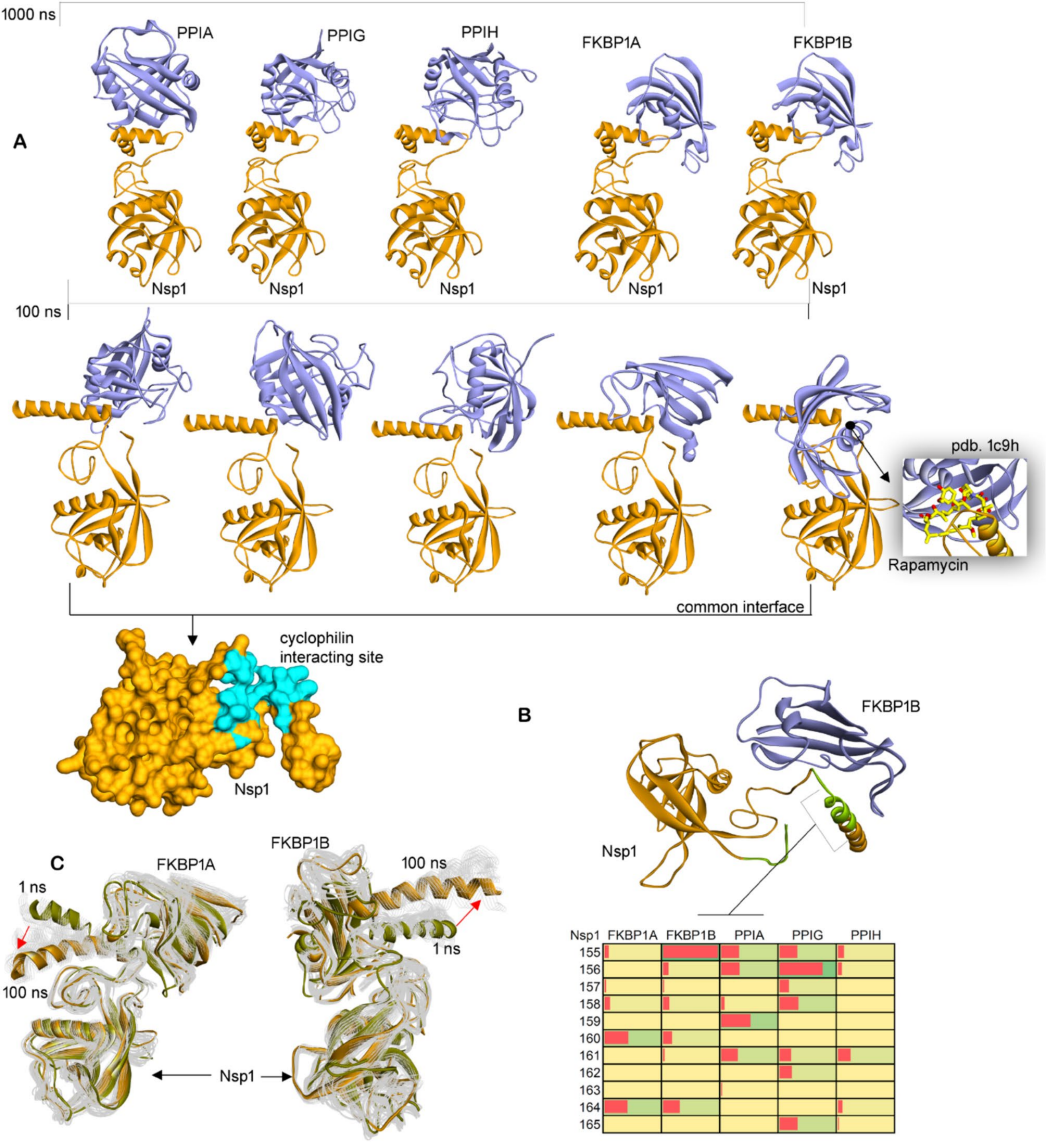
The cyclophilins–Nsp1 complexes along with their binding pockets. **A** Cyclophilins screened against Nsp1. The PPIA (pdb id.: 1ak4 (Gamble et al. 1996)), PPIG (pdb id.: 2gw2 (Davis et al. 2010)), PPIH (pdb id.: 1mzw (Reidt et al. 2003)), FKBP1A (pdb id.: 1a7x (Schultz and Clardy 1998)), and FKBP1B (pdb id.: 1c9h (Deivanayagam et al. 2000)) cyclophilins were evaluated. The right panel demonstrate the FKBP1B protein with the Rapamycin drug in yellow (Deivanayagam et al. 2000), and the bottom panel describes Nsp1 surface view along with common cyclophilin interacting sites in blue. **B** High occupancy interacting residues from cyclophilins and SARS-CoV-2 Nsp1. Boxes with red color describe frequency of individual interactions (Table S2), and residues ranging 155–165 aa from Nsp1 were found interacting with the cyclophilins. Over the Nsp1–FKBP1B structures, the residues of Nsp1 found interacting with cyclophilins are labeled in green. **C** Conformational changes of Nsp1 with respect to FKBP1A- or FKBP1B-binding partners over the MD simulation time course

High-throughput yeast two-hybrid (Pfefferle et al. 2011; Semper et al. 2021) technique has revealed redundant interactions between SARS-CoV Nsp1 and a group of host proteins with peptidyl-prolyl cis–trans-isomerase activity, involving the cyclophilins or immunophilins (Vankadari and Ghosal 2024). These components modulate the Calcineurin/NFAT pathway which has an important role in immune cell activation (Feske et al. 2001; Hogan et al. 2003). Knockdown PPIA in Caco-2 cells shall block the replication of HCoV-NL63 (Carbajo-Lozoya et al. 2014). Therefore, we explored the crucial PPI pattern between SARS-CoV-2 Nsp1 and cyclophilins (PPIA, PPIG, PPIH, FKBP1A, or FKBP1B). Nsp1 with cyclophilins formed a well-defined helical C-terminus and a tilted C-terminal region at the S166 site (Fig. 2A). Individual cyclophilins bind to the conserved loop or linker region, connecting the C-terminal and central regions (Figs. 1A, 2A). In particular, the leader protein from the FKBP1B–Nsp1 complex docks the cyclophilin in the active site region required for Rapamycin binding (Fig. 2A).

**Fig. 3:**
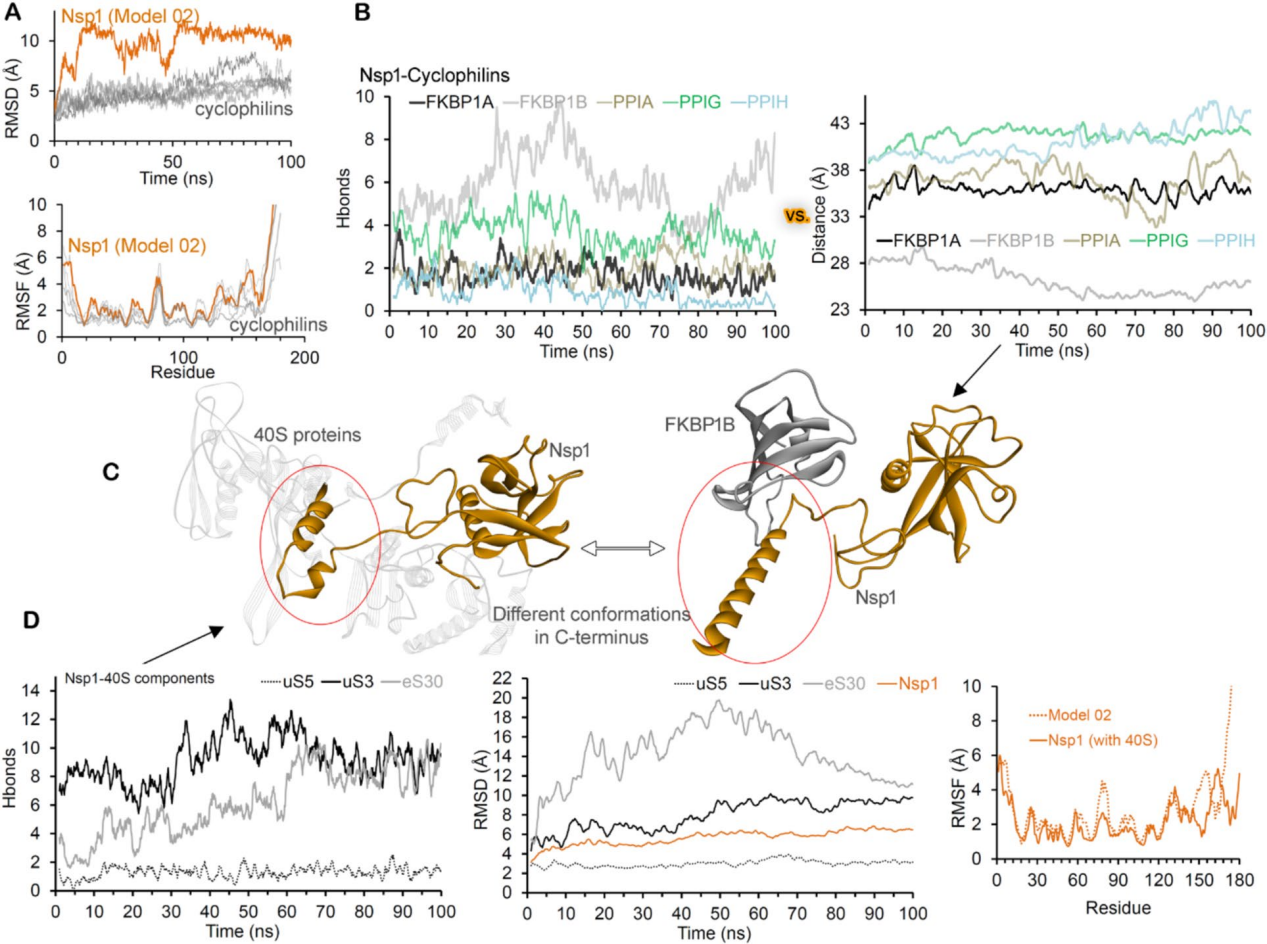
Protein–protein interactions of Nsp1 with cyclophilins and 40S ribosomal components. **A** The RMSDs (Root-Mean-Square Deviations) of SARS-CoV-2 Nsp1 compared when bound with FKBP1A, FKBP1B, PPIA, PPIG, and PPIH cyclophilins. The bottom panel represents RMSFs (Root-Mean-Square Fluctuations) of Nsp1 in different complexes or unbound state. **B** Nsp1–cyclophilins intermolecular interactions compared with change in the distance center of mass between Nsp1 and cyclophilins. **C** Distinct C-termini conformation of Nsp1 with FKBP1B and 40S ribosome. **D** The uS5, uS3, and eS30 components from 40S ribosome interact with Nsp1 (pdb id.: 6zoj (Schubert et al. 2020))

The apo-form of Nsp1 structure was found to gain a conformational switch at its C-terminus (Figs. 1, S3 and S4), and therefore, we considered both tilt and non-tilt Nsp1 C-terminal conformation to screen against cyclophilins. Structural stability of Nsp1 based on RMSD (root-mean-square deviation) and RMSF revealed that the presence of cyclophilins stabilizes the leader Nsp1 protein (Figs. 3A and S4). Nsp1 in its unbound form displays a higher RMSD reaching up to ~ 10 Å, whereas this value was found to be ~ 5 Å when bound with cyclophilins (Fig. 3A). Monitoring individual residue flexibility (RMSFs; Fig. 3B) of Nsp1 in different conditions demonstrated that the central domain is more stable compared to terminal regions, and the higher RMSDs in the unbound form of Nsp1 emerged from the flexibility in terminal regions.

The PPIs of Nsp1 networking proteins suggest that FKBP1B has formed a high number of interactions and PPIH lowest interactions among different cyclophilins (Fig. 3B). These findings correlate the distance center of mass between Nsp1-cyclophilins, as the distance is reduced, it enhances PPIs forming a high-frequency pharmacophore (Table S2, Fig. 3C). Nsp1 residues making high-frequency PPIs with cyclophilins (Fig. 2B and Table S2) originated from the 155–165 aa (C-terminal) region, and FKBP1B with best affinity for Nsp1 was found mainly interacting with the C-terminal site (Fig. 2B). Later, the PPIG among cyclophilins was found interacting with both N- and C-termini regions of Nsp1 (Fig. 2B). The E155 and D156 residues (Nsp1) were found making high occupancy interactions with FKBP1B and with PPIG, respectively (Fig. 3B and Table S2). The C-terminus domain of Nsp1 has different conformation toward different cyclophilins, e.g., with FKBP1A and FKBP1B, it showed distinct displacements (Fig. 2C). PPIH cyclophilin was found to make the least interaction with Nsp1, which could be the cause of folding of the C-terminus toward the central domain (Fig. S5). Overall, the cyclophilins were found in high-affinity binding states with Nsp1 close to the region where the C-terminus bends when interacting with the 40S ribosomal subunit (Schubert et al. 2020).

### Pharmacophore of Nsp1 with 40S ribosomal proteins

The Nsp1 was reported interacting with the 40S ribosomal subunit interfering with mRNA binding, and the cryo-EM (pdb id.: 6zoj (Schubert et al. 2020)) structure revealed that its C-terminus docks to uS5, uS3, and eS30 components. These Nsp1–40S interactions are suggested to be site specific formed by a set of conserved residues (Schubert et al. 2020). The cryo-EM structure (pdb id.: 6zoj (Schubert et al. 2020)) consists of only the C-terminal Nsp1 domain, and therefore, we evaluated the full-length Nsp1 structure networking with the 40S components exploring their dynamics (Fig. S6). To generate the Nsp1–40S model complex, the energy-minimized Nsp1 full-length structure was replaced with the fragmented C-terminus structure in the 40S ribosome subunit (pdb id.: 6zoj (Schubert et al. 2020)), mainting the conformations required for interactions with uS5, uS3, and eS30.

Investigating the stability of the Nsp1 in the apo-form (unbound state) and complexed with 40S ribosomal proteins suggests that the presence of 40S components stabilizes the Nsp1 residues (C-terminal domain; Fig. 3D). The Nsp1 protein copies after initial fluctuations were found to be stable by the end (~ 80 ns) of the MD simulation (Fig. 3D). Among the 40S components, the eS30 was found to be unstable during the initial simulation time, and a correlating behavior was observed in terms of Nsp1–eS30 interactions (Fig. 3D). Though showing differences in the initial number of PPI, both uS3 and eS30 formed an equivalent number of interactions by the end of the MD simulation (Fig. 3D). Despiste high stability of uS5, it formed the least number of PPIs with Nsp1 as compared to uS3 or eS30 (Figure S6D). Analyzing different conformational states of Nsp1 C-terminal structure with cyclophilins or ribosome components over the MD simulation timescale, it could be highlighted that C-termini region forms a bended position only when bound with the 40S ribosome despite cyclophilins docking to the same location (Fig. 3C, D).

The uS3 gene has formed a higher number of interactions with the Nsp1 showing a similar trend in residues with high-frequency occupancy (Figs. 3D and S6A). A higher number of residues from the Nsp1 protein interacting with the 40S ribosomal components (uS5, uS3, and eS30) resides within its C-terminal region. Mapping the cryo-EM (Schubert et al. 2020) and cross-linking mass spectrometry datasets (Slavin et al. 2021), it was demonstrated that Nsp1 can interact with uS3 ribosomal protein at several sites (Sakuraba et al. 2022). In our MD simulations, apart from C-terminus hotspots, a set of binding regions from the central domain region were observed, which highlighted that uS3 can also interact with the Nsp1 central domain (Fig. S6A). The Nsp1 residues, such as E41, E87, E155, D156, E159, E176, and N178, were found making long lasting interactions with the 40S ribosomal components (Fig. S6A). In addition, R171 and R175 from Nsp1 bound with eS30 were found in common with the cryo-EM studies (Schubert et al. 2020). The Nsp1 showed stable conformational dynamics over the simulation time course, and in particular, the linker region connecting C-terminal and central domain showed significant stretching within its structure (Fig. S6B).

### Protein–protein interactions derived motifs as modulator for Nsp1

Nsp1 has been reported with the ability to alter host RNA processing and degradation, and one of the mechanisms predicted is through altering the intermolecular network in the nuclear RNA exosome complex (Jonasson 2020). Exosome eliminates RNAs that result from the cryptic transcription, and is hence crucial in RNA quality control mechanism (Kilchert et al. 2016; Wlotzka et al. 2011; Pefanis et al. 2015). Due to the similar structural orientation of SARS-CoV-2 Nsp1 and exosome Rrp46 protein (Figure S1D), it was speculated that the Nsp1 may also bind the Rrp45 mimicking Rrp45–Rrp46 interactions (requires further validation) (Jonasson 2020). However, the Nsp1 interactions with the exosome have been reported earlier with Dis3/Rrp44 (Bujanic et al. 2022) and Exosc3/Rrp40 in yeast (Gerassimovich et al. 2021). Our docking results suggest a similar orientation for the Nsp1-Rrp45 which resembles that of the Rrp45–Rrp46 complex (Fig. 1D). Investigating the dynamics of Nsp1 bound and unbound with Rrp45, highlighted that the presence of Rrp45 stabilizes the SARS-CoV-2 protein (Figs. S7A, S7B and S8). The unbound Nsp1 displays higher RMSDs during the initial time course and significant flexibility was observed in its C-terminus (Fig. S7B). Over the 1 µs simulation, the Nsp1 with Rrp45 has obtained a structural displacement from the initial position similar to the Rrp46 with Rrp45 (Fig. S1C). Though Nsp1 and Rrp46 formed different conformations, they shared a common interface with Rrp45, and Nsp1 has formed a higher number of interactions with Rrp45 (Figure S7C). Such a higher number of interactions could be the cause of a tilt movement in the C-terminal region of Nsp1 (Fig. S7C). The E81 residue from Rrp45 formed a high-frequency conserved interaction with the R77 (Nsp1) and R81 (Rrp46) from the Rrp45–Nsp1 and Rrp45–Rrp46 complexes, respectively (Figure S7). For the Rrp45–Nsp1 system, several interactions from Nsp1 reside in the central domain.

To mimic the interaction of the Nsp1–Rrp45 or block the activity of Nsp1, a set of Rrp45-derived PPIs linear motifs or peptides derived were screened against the two distinct predicted Nsp1 active sites (Figs. S7D and S8). These active sites predicted over our modeled Nsp1, corresponding to the pockets revealed from unbiased simulations (Borsatto et al. 2022; Ma et al. 2022). Self-derived peptides from a human gene can become the basis to design peptide modulators against Nsp1 (Brankiewicz et al. 2023). During initial screening of peptides with both Nsp1 predicted sites at the N- and C-terminus suggest that pep06, pep12, pep13, and pep14 (Fig. S7) formed high-affinity binding. In addition, pep08 and pep01/pep05 peptides were found making high binding affinity toward the N-ter and C-ter active sites, respectively (Figs. S7 and S9). High affinity binding pep12 (TAFKMEKAPIDTSDVEEKA), pep14 (EEIIAEAEPP), and pep13 (IDTSDVEEKA) peptides were further investigated with Nsp1 to ensure their obtained conformation when placed in the solvent environment (Fig. S9).

**Fig. 4:**
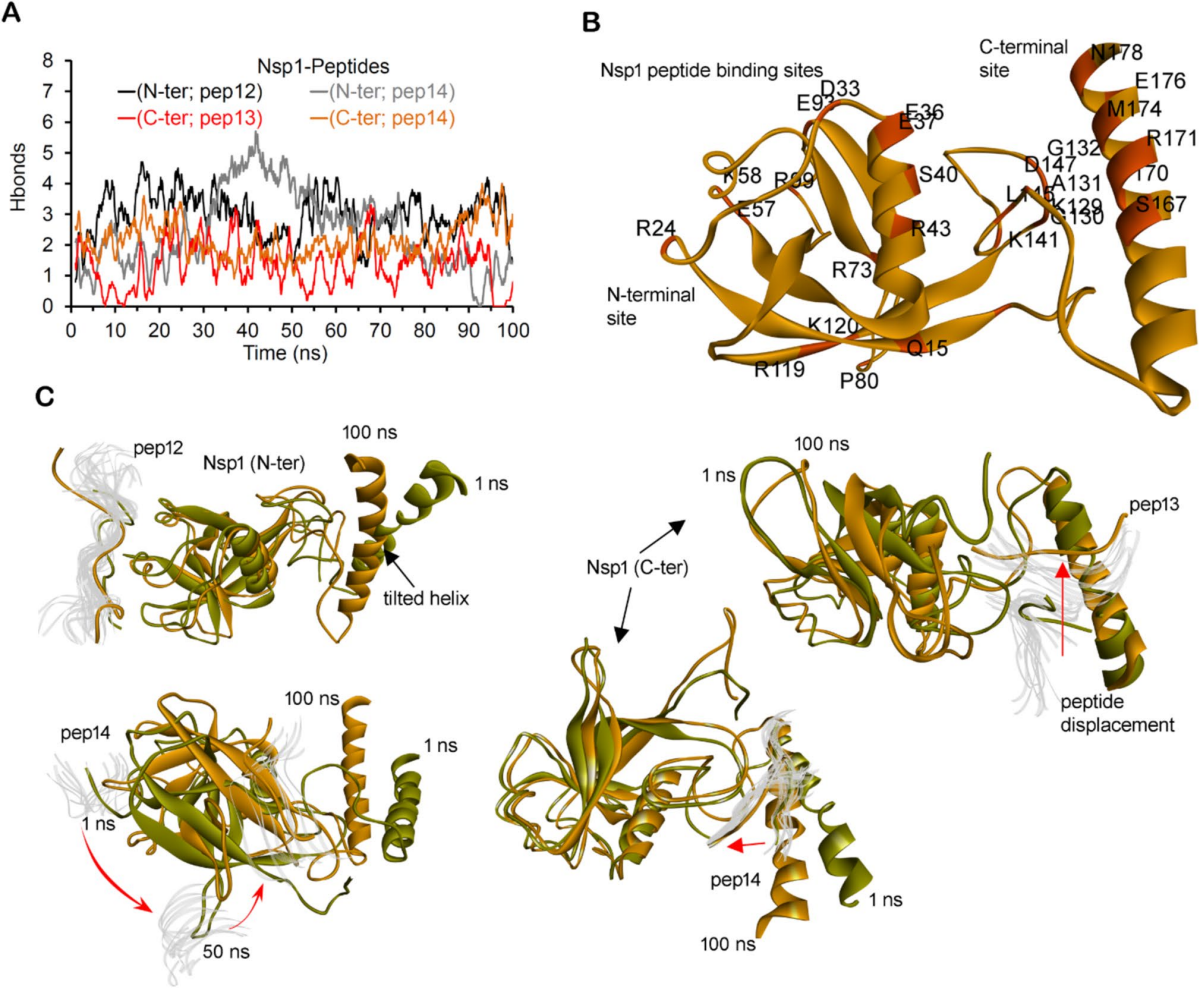
Self-derived peptides (hRrp45) against two distinct predicted active sites of SARS-CoV-2 Nsp1. **A** Intermolecular hydrogen-bonding interactions between the Nsp1-peptides (pep12; TAFKMEKAPIDTSDVEEKA, pep13; IDTSDVEEKA, and pep14; EEIIAEAEPP). **B** High occupancy interactions displayed over the Nsp1 structure. **C** The conformational dynamics of the peptides and Nsp1 over the MD simulation

Investigating the stability change over the time course, it was observed that diverse length peptides showed contradictory behavior with Nsp1 (Fig. S10). The pep14 peptide binding with the N-terminal site has induced stability in Nsp1, whereas the same peptide when binding the C-terminus destabilizes the protein (Fig. S10). Many peptides complexed with Nsp1 have RMSDs of ~ 5 Å, but exceptionally the longer in length (pep12) peptides have high flexibility (Fig. S10). The pep12 and pep14 peptides have formed a stable number of PPIs and the residue making high-frequency networks originate from the linker region close to the C-terminus (Fig. 4A, B, Table S3). A set of Nsp1 residues from the α-helical regions from the central and C-terminal domain were found interacting with the peptides. Visualizing conformational dynamics of peptides with Nsp1, it was observed that peptides were displaced from their initial position over the Nsp1 structure making high-frequency interactions with other regions (Fig. 4B, C). The pep14 molecule interacting with the N-terminal site during the initial time frame has shown a significant displacement movement toward the C-terminal site (Fig. 4C).

### Adopted conformations of the C-terminal region from SARS-CoV-2 Nsp1

**Fig. 5:**
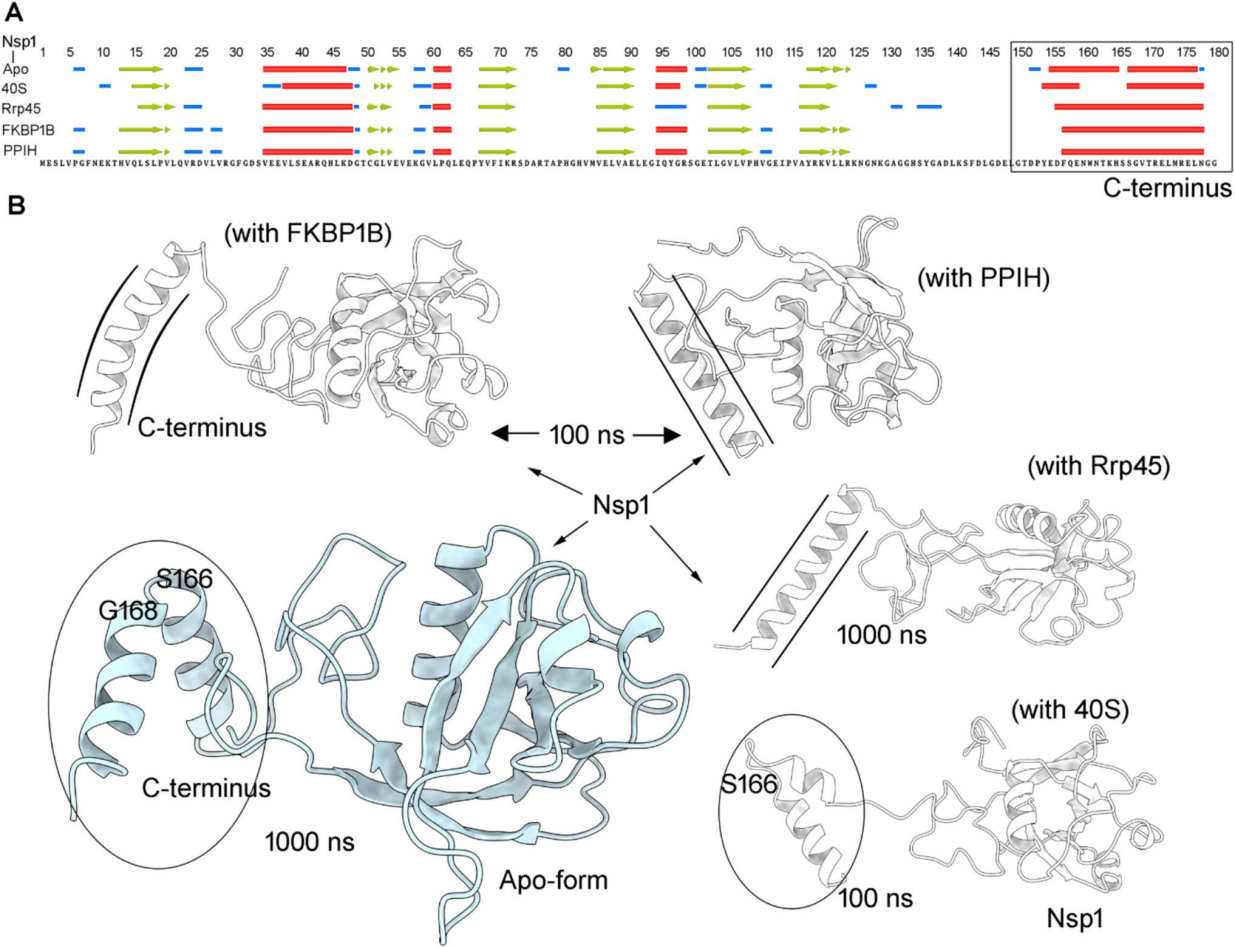
Plasticity of the SARS-CoV-2 Nsp1 C-termini. **A** Secondary structure gained by SARS-CoV-2 Nsp1 by the end of MD simulation time course (β-sheet is presented in arrow and α-helical region as tubes).** B** Adapted conformations within the α-helical C-terminal region from the Nsp1 protein, which is mediated according to the complex it interacts with; cyclophilins or 40S ribosomal subunit, or RNA exosome. Individual Nsp1 coordinates were retrieved from the end of the MD simulation time, and conformational changes are highlighted with lines

Physicochemical properties of different protein’s plasticity with Nsp1 were explored using MD simulation, shedding light on the functional implication of interactions with the leader protein. The MD simulated trajectories revealed that the leader protein adapted stable structural folding or conformations with respect to its interacting cyclophilins, the 40S ribosomal subunit, and exosome components (Fig. 5). Such features of structural adaptation within the Nsp1 protein enhanced its protein–protein/peptide hydrogen-bond interactions. In the unbound or apo-form system, the Nsp1 C-terminus gained a bent (S166-G168 aa) conformation by the end of 1 µs MD simulation; such a state is suggested to be crucial to interact with the 40S ribosome subunit (Fig. 5). A similar conformation of Nsp1 as with the 40S complex (Schubert et al. 2020) was obtained at an extensive 1 µs simulation time for the unbound state or apo-form. Analyzing secondary structure of SARS-CoV-2 Nsp1 retrived from the end of the simulation time course demonstrated a split in the α-helical C-terminal region when with 40S components and apo-form (Fig. 5).

From the Nsp1–FKBP1B complex, the C-terminal helix formed a moderate tilt movement that originated at a site close to residue S166 (Fig. 5). It has been reported that an alteration within the 160–173 aa region of SARS-CoV Nsp1 leads to the inactivation of the protein affecting its ribosome-dependent functions (Narayanan et al. 2008). Considering this fact, it could be speculated that K164A and H165A mutants inactivating the Nsp1 protein may have hindered the conformational switch of the C-terminal region (Fig. 5). The C-terminal domain of the Nsp1 with PPIH or Rrp45 showed their movements in opposite directions (Fig. 5), and in particular, it was displaced toward the central domain when complexed with PPIH (Fig. 5). A similar tilt conformation was observed in the previous simulated Nsp1 protein (Sakuraba et al. 2022). Furthermore, the S40, R43, A131, K120, and K141 residues were found in common between our observed hydrogen-bonding interactions and those seen in the previous studies (Sakuraba et al. 2022; Slavin et al. 2021).

## Materials and methods

### Ab initio modeling of the Nsp1 and its partner proteins

Due to the lack of a complete structure for SARS-CoV-2 Nsp1, the crucial step was to generate a 3D model of the leader protein. Using the available Nuclear Magnetic Resonance (NMR) structures of the SARS-CoV Nsp1 (pdb id.: 2gdt and 2hsx (Almeida et al. 2007), with missing C-terminus) as a template, the full-length Nsp1 was built. The models were generated using a web-based full-chain protein prediction server; I-TASSER tool (Roy et al. 2010). I-TASSER is a comprehensive and automated pipeline for protein tertiary structure modeling; it scans a representative pdb library to search for possible folds using multiple threading alignments and reassembles the complete model together using iterative structure assembly simulations (Roy et al. 2010). Using this integrated platform, 6 models for Nsp1 were obtained (five best-resulted models and the nsp1 model already available in I-TASSER). All six structural models of nsp1 protein obtained from the I-TASSER were subjected to 100 ns MD simulations.

MD simulations of the modeled systems were performed using the GROMACS 4.6.5 (Pronk et al. 2013) package and applying the CHARMM27 forcefield (Bjelkmar et al. 2010). Previously studies have reported that CHARMM27 can be used to investigate the dynamics of non-structural proteins (Nsp1), validating in silico findings using biochemical assays (Nsp1 moiety being from Dengue virus) (Songprakhon et al. 2020). All the systems were solvated by a single-point charge model in a dodecahedron box with a minimum of 10 Å edge distance and periodic boundary conditions (PBC) were adopted in all directions. The Na + Cl- corresponding to a concentration of 150 mM were added to mimic the cellular environment and produce neutral systems. To begin with, the steepest descent method was used to minimize the total potential energy of each system with the equilibration time step set to 50,000. The long-range electrostatic interactions were calculated using the particle mesh Ewald (PME) method (Darden et al. 1993), and the bonds containing hydrogen atoms were treated using the LINCS (LINear Constraint Solver) algorithm (Hess et al. 1997). Coulomb (electrostatic) and van der Waals cutoffs were set to 10 Å. The energy-minimized systems were subsequently equilibrated with NPT [number of particles (N), system pressure (P), and temperature (T); isobaric-isothermal] ensemble simulation for 1000 ps. The standard temperature and pressure (300 K and 1 bar) were coupled with V-rescale thermostat (Bussi et al. 2007) and Parrinello-Rahman barostat (Parrinello and Rahman 1981) methods, respectively. These equilibrated systems were used to perform the final MD production runs. A leap-frog integrator (Gunsteren and Berendsen 1988) was used to integrate the equations of motion, while a time interval of 10 ps was used to save the trajectories for further analysis. The production run was performed for all systems and analysis was carried out using the GROMACS and visual molecular dynamics (VMD) (Humphrey et al. 1996) tools (Table S1). Protein representations were prepared in the BIOVIA Discovery Studio visualizer (Dassault Systèmes, BIOVIA Corp., San Diego, CA, USA), ChimeraX (Meng et al. 2023), and Molecular Operating Environment (MOE; Chemical Computing Group Inc., Montreal, QC, Canada) pipelines.

The Nsp1 average structure was extracted from each of the simulated systems, and models consisting of regions of local ordered secondary structure (α-helices and β-sheets) that were characterized by the local rotational state of the protein backbone, specified by the two dihedral angles *ϕ* and *ψ* (Mannige et al. 2016). The secondary structure analysis was performed for the averaged models of Nsp1 from MD simulation using a Ramachandran plot, enabling immediate assessment of the geometric nature of protein (Mannige et al. 2016). In the initial 100 ns of MD simulation, the model number #2 of Nsp1 showed well-defined secondary structures (α-helices and β-sheets). The structures obtained at different time frames for Nsp1 (model #2) were further considered to investigate with different interacting components (Table S1).

### Protein or peptide screening against Nsp1

A set of cyclophilin protein structures (PPIA (pdb id.: 1ak4 (Gamble et al. 1996)), PPIG (pdb id.: 2gw2 (Davis et al. 2010)), PPIH (pdb id.: 1mzw (Reidt et al. 2003)), FKBP1A (pdb id.: 1a7x (Schultz and Clardy 1998)), and FKBP1B (pdb id.: 1c9h (Deivanayagam et al. 2000)) were retrieved from the protein data bank (www.rcsb.org). Individual cyclophilin proteins were screened against the energy-minimized SARS-CoV-2 Nsp1 protein, using the ZDOCK web server (Pierce et al. 2014). ZDOCK (https://zdock.umassmed.edu/) optimizes a global docking search on a three-dimensional grid using a Fast Fourier Transform algorithm (FFT) with shape complementarity, electrostatics, and statistical potential terms for scoring the complex structures (Pierce et al. 2014; Chen and Weng 2002). For generating the Nsp1–40S ribosome complex, our energy-minimized Nsp1 full-length retrieved from MD simulation of its unbound form was used to replace the fragmented C-terminus structure in the 40S ribosome subunit (pdb id.: 6zoj (Schubert et al. 2020)). Maintaining Nsp1 conformation with the 40S ribosome proteins the MD simulation was performed for 100 ns, using the GROMACS 4.6.5 (Pronk et al. 2013) packge and applying the CHARMM27 forcefield (Bjelkmar et al. 2010)).

The crystal structure of Rrp45 (RNA exosome complex; pdb id.: 2nn6 (Liu et al. 2006)) has missing residues or regions that were built by an automated protein homology modeling server (SWISS-MODEL (Arnold et al. 2006)). This workspace (https://swissmodel.expasy.org/) is a web-based integrated service which uses the protein sequence and a three-dimensional structure having a high enough similarity to the sequence (Arnold et al. 2006). To reduce computational time, the SWISS-MODEL was used to generate missing residues in Rrp45. Over the docked Rrp45-Rrp46 and Rrp45-Nsp1 (retrieved from ZDOCK), the MD simulation was performed for 100 ns using GROMACS 4.6.5 (Pronk et al. 2013) and applying the CHARMM27 forcefield (Bjelkmar et al. 2010). The structures extracted from the Nsp1–40S/RNA exosome/cyclophilin complexes were examined individually in the Discovery Studio Client v18.1 (Dassault Systemes, BIOVIA Corp., San Diego, CA, USA) program besides analyzing their PPIs.

Over the Nsp1 retrieved from MD simulations, the active sites were predicted using the alpha shapes technique site finder module within the MOE package (Chemical Computing Group Inc.). The site finder option calculates the possible active sites in the 3D atomic coordinates of the receptor, and determines potential sites for docking of ligand binding (Goodford 1985). Using this protocol, two different active sites (labeled as N- and C-termini) were obtained over the SARS-CoV-2 Nsp1 structure. The peptides with a sequence derived from the binding region (amino acids) of Rrp45 were modeled using the protein builder module from the MOE software package (Chemical Computing Group Inc.) (Kitchen et al. 2004). Resulting peptide structures were subjected to energy minimization using the CHARMM27 forcefield (Bjelkmar et al. 2010), which computes the local minima of coordinates using a potential energy function. Other parameters were non-bonded cutoff 8 Å and 10 Å, rigid water molecules constraints, and RMS gradient to 0.1. Following structure preparation and energy minimization, the peptides were screened against the Nsp1. Such screening was performed with the ‘rigid receptor’ protocol and the peptide was allowed to be flexible (rotatable bonds). In Nsp1-peptide docking carried out using the ‘Triangle matcher’ placement method, a set of 1000 peptide conformations were generated. All these conformations per peptide were scored by a forcefield-based scoring function, GBVI/WSA dG (kcal/mol), which determines the binding free energy of the peptide from a given pose (Corbeil et al. 2012; Wojciechowski and Lesyng 2004). Furthermore, the best conformations of peptides having highest binding affinity toward Nsp1 were subjected to MD simulations to understand their dynamics. For the Nsp1–peptide complexes (pep12; TAFKMEKAPIDTSDVEEKA, pep13; IDTSDVEEKA, and pep14; EEIIAEAEPP), the 100 ns MD simulations were performed using GROMACS 4.6.5 (Pronk et al. 2013) and applying the CHARMM27 forcefield (Bjelkmar et al. 2010).

## Conclusions

Nsp1 acts in multiple ways toward the host cell, triggering host cell mRNA cleavage and decay, as well as inducing cytokines and chemokines. SARS-CoV-2 couples global inhibition of translation through Nsp1 with efficient translation of the viral mRNA to allow expression of viral genes. Any alteration in the C-terminus of Nsp1 led to the inactivation of the protein, and therefore, considering the crucial role of this gene in different complexes, we studied full-length protein and its protein network with cyclophilins, the 40S ribosome subunit, and RNA exosome. During 1000 ns of MD simulation, the Nsp1 formed a well-defined secondary structure and the hydrophilic (C-terminus) surface demonstrated significant fluctuations. Screening of cyclophilins with energy-minimized SARS-CoV-2 Nsp1 revealed that among different studied cyclophilins, the FKBP1B has high and the PPIH has least affinity with Nsp1. Such a wide difference in the interaction pattern with Nsp1 could be the cause of structural folding within the C-terminus toward the central domain. In particular, the FKBP1B mainly interacts with the C-terminus site of Nsp1, whereas the PPIG was found interacting with different regions. Nsp1 residues E155 and D156 were found making high occupancy interactions with FKBP1B and with PPIG, respectively. The Nsp1 protein was found interacting with the FKBP1B in the Rapamycin drug-binding pocket. The distance center of mass between Nsp1-cyclophilins suggests that as the distance is reduced, it enhances PPIs. Frequently, the Nsp1 residues from the 155–165 range were involved in the interactions with the cyclophilins. The C-terminus domain of Nsp1 has a different conformation toward different cyclophilins, and in particular, the leader protein with FKBP1A and FKBP1B showed opposite conformations. The majority of cyclophilins bind to the conserved Nsp1 loop or linker region joining the C-terminal and central domain.

The uS3 and eS30 proteins from the 40S ribosome subunit formed an equivalent number of interactions with Nsp1, and the C-terminal regions of Nsp1 were found stable when complexed with the 40S subunit components. The binding sites from the Nsp1 protein with the 40S ribosome components (uS5, uS3, and eS30) were found residing in the C-terminal region with some regions from the central domain region. R171 and R175 amino acids from Nsp1 bound with eS30 were found in common with the cryo-EM studies. A dataset of Rrp45-derived linear peptide motifs screened against the SARS-CoV-2 Nsp1 protein suggests that diverse length peptides have shown distinct behavior. Pep14 (EEIIAEAEPP) peptide binding with the N-terminal site has induced stability in Nsp1, whereas the same peptide has shown the contrary effect when binding with the C-terminal site. Based on the activity of these peptides, it could be proposed that they may act as a blocker or modulator for the Nsp1 protein from the SARS-CoV-2.

Classical MD simulations revealed the physicochemical properties of different protein’s plasticity with Nsp1, as well as shed light on the functional implications of this plasticity in the interaction with SARS-CoV-2 leader protein. Comparing distinct structural changes within the Nsp1 C-terminal region with cyclophilins or ribosome, it was observed that the C-terminal region bends in apo-form and when bound with the 40S ribosome. Such bending of the Nsp1 (apo-form hinge in the α-helical C-terminal region) occurs at a site close to S166 residue, which is crucial for positioning of the protein to the 40S ribosome. Identifying key interacting residues within different Nsp1-cyclophilins or 40S ribosome or RNA exosome complexes, our findings revealed that SARS-CoV-2 Nsp1 protein has a versatile C-terminus region, which could change its conformations with respect to its binding network. These findings suggest that the Nsp1 protein has novel binding sites which may aid future drug discovery programs aimed at targeting the coronavirus family.

## Supplementary Information

Below is the link to the electronic supplementary material.

Supplementary file1 (PDF 2700 KB)

Supplementary file2 (MP4 6249 KB)

## Data Availability

The data supporting this study’s findings are available in the article and its supporting materials.
