# Supplementary material for: Structural adaptability of SARS-CoV-2 Nsp1 with the host network
Source: Eur Biophys J. 2025 Jun 14;55(1):41–54. doi: 10.1007/s00249-025-01762-y (PMC12929329; doi:10.1007/s00249-025-01762-y)
Supplement: Supplementary file 1 — Supplementary file1 (PDF 2700 KB) [file 249_2025_1762_MOESM1_ESM.pdf]

Structural adaptability of SARS-CoV-2 Nsp1 with the host network

Monikaben Padariya[1,*], Ted Hupp[1,2], Umesh Kalathiya[1,*]

[1]International Centre for Cancer Vaccine Science, University of Gdansk, ul. Kładki 24, 80-822, Gdansk, Poland
[2]Institute of Genetics and Molecular Medicine, University of Edinburgh, Edinburgh, Scotland EH4 2XR, United Kingdom
* Correspondence: monikaben.padariya@ug.edu.pl (M.P) and umesh.kalathiya@ug.edu.pl (U.K)

Supporting Materials

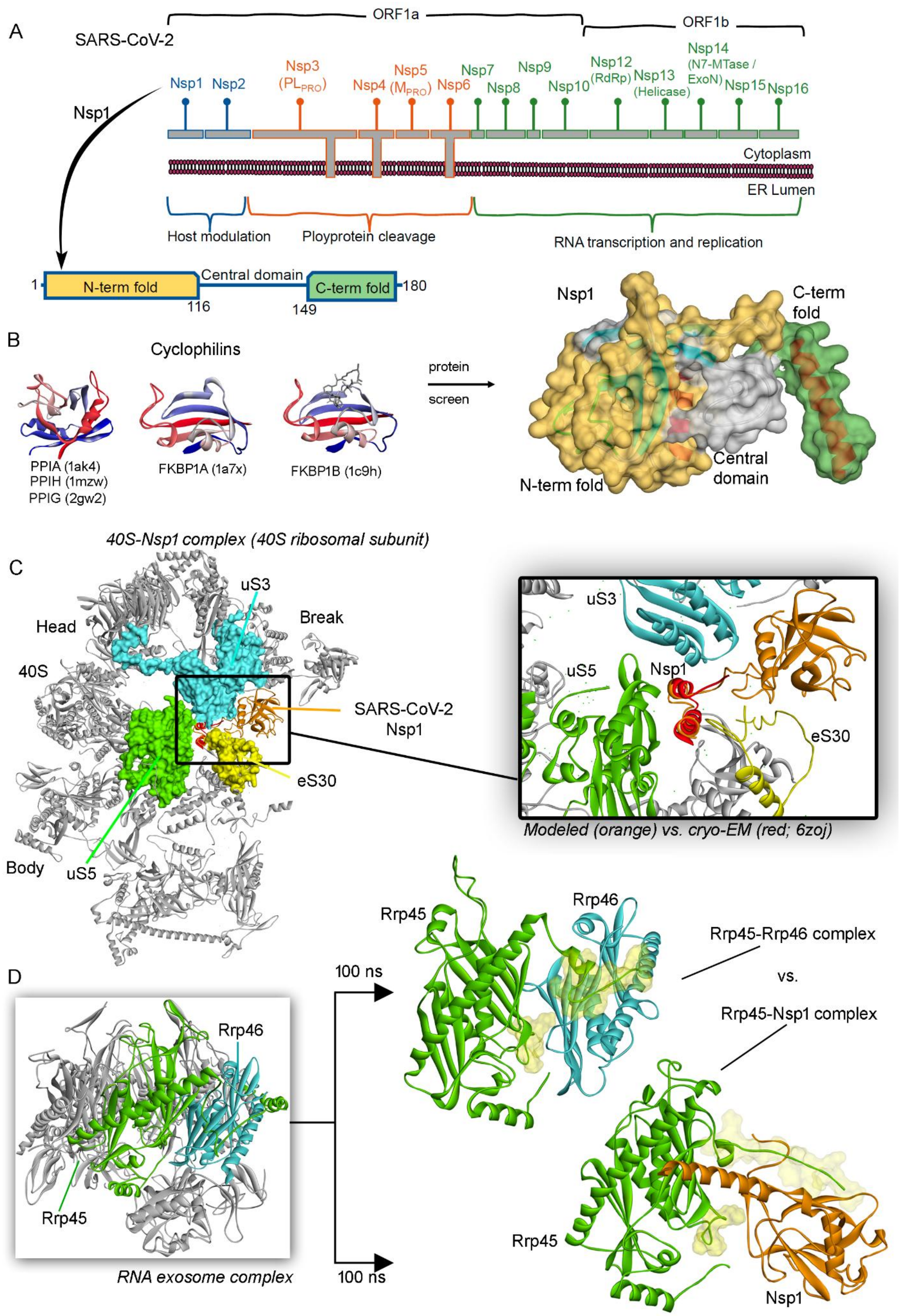

Figure S1. The non-structural leader protein (Nsp1) protein. **(A)** The genomic structure of the virus codes for 16 non-structural proteins (Nsp1-16 merged as a polyprotein ORF1). The bottom diagram presents different domains of SARS-CoV-2 Nsp1 protein, and the right panel describes domains over the protein structure. **(B)** The cyclophilins docked or screened against the SARS-CoV-2 Nsp1 protein. The cyclophilins considered in this work were; PPIA (pdb id.:

1ak4), PPIG (pdb id.: 2gw2), PPIH (pdb id.: 1mzw), FKBP1A (pdb id.: 1a7x), and FKBP1B (pdb id.: 1c9h). **(C)** Nsp1 conformation with the 40S ribosomal subunit, studied applying our molecular dynamic simulation approach (MDS; pdb id.: 6zoj). For the Nsp1-40S complex, our optimized Nsp1 full structure (in orange) was replaced with the fragmented C-terminus structure in the 40S ribosome cryo-EM (cryogenic electron microscopy) structure (pdb id.: 6zoj; in red). **(D)** The left panel represents eukaryotic RNA exosome consisting of six RNase proteins (Rrp41, Rrp42, Rrp43, Rrp45, Rep46, and Mtr3; pdb id.: 2nn6) and three RNA binding proteins. Nsp1 / Rrp46 were investigated to trace binding efficiency with the Rrp45 (from the exosome complex). The right panel shows the docked Nsp1 with Rrp45 (after 100 ns of MDS), and the binding surface is represented in yellow compared with the Rrp45-Rrp46 complex.

Table S1. Outline of different MDS systems studied, applying CHARMM27 forcefield and GROMACS package. The systems were solvated by a single-point charge model in a dodecahedron box with a minimum of 10 Å edge distance and periodic boundary conditions were adopted in all directions. The Na+Cl- corresponding to a concentration of 150 mM were added to mimic the cellular environment and produce neutral systems.

Systems	***Nsp1 (bound - unbound models)***		
	**No.**	**Amino acid**	**MDS***
1	Model #1 (apo-form)	1-180 aa	100 ns
2	Model #2 (apo-form)	1-180 aa	1000 ns
3	Model #3 (apo-form)	1-180 aa	100 ns
4	Model #4 (apo-form)	1-180 aa	100 ns
5	Model #5 (apo-form)	1-180 aa	100 ns
6	Model #6 (apo-form)	1-180 aa	100 ns
	**Cyclophilins**		
7	Nsp1 (model #2)-FKBP1A	pdb id.: 1a7x, 1-107 aa	100 ns
8	Nsp1 (model #2)-FKBP1B	pdb id.: 1c9h, 1-107 aa	100 ns
9	Nsp1 (model #2)-PPIA	pdb id.: 1ak4, 1-165 aa	100 ns
10	Nsp1 (model #2)-PPIG	pdb id.: 2gw2, 7-179 aa	100 ns
11	Nsp1 (model #2)-PPIH	pdb id.: 1mzw, 5-177 aa	100 ns
	40S ribosome components		
12	Nsp1(model #2)-40S(uS5, uS3, and eS30)	pdb id.: 6zoj	100 ns
	**The exosome complex**		
13	Rrp45 (apo-form)	pdb id.: 2nn6, 1-302 aa	1000 ns
14	Rrp46 (apo-form)	pdb id.: 2nn6, 25-235 aa	1000 ns
15	Nsp1 (model #2)-Rrp45	Rrp45; pdb id.: 2nn6	1000 ns
16	Rrp46-Rrp45	pdb id.: 2nn6	1000 ns
	**Rrp45 derived peptides**		
17	Nsp1 (model #2; N-ter)-pep12	pep12; TAFKMEKAPIDTSDVEEKA	100 ns
18	Nsp1 (model #2; C-ter)-pep13	pep13; IDTSDVEEKA	100 ns
19	Nsp1 (model #2; N-ter)-pep14	pep14; EEIIAEAEPP	100 ns
20	Nsp1 (model #2; C-ter)-pep14	pep14; EEIIAEAEPP	100 ns

*MDS, molecular dynamics simulations.

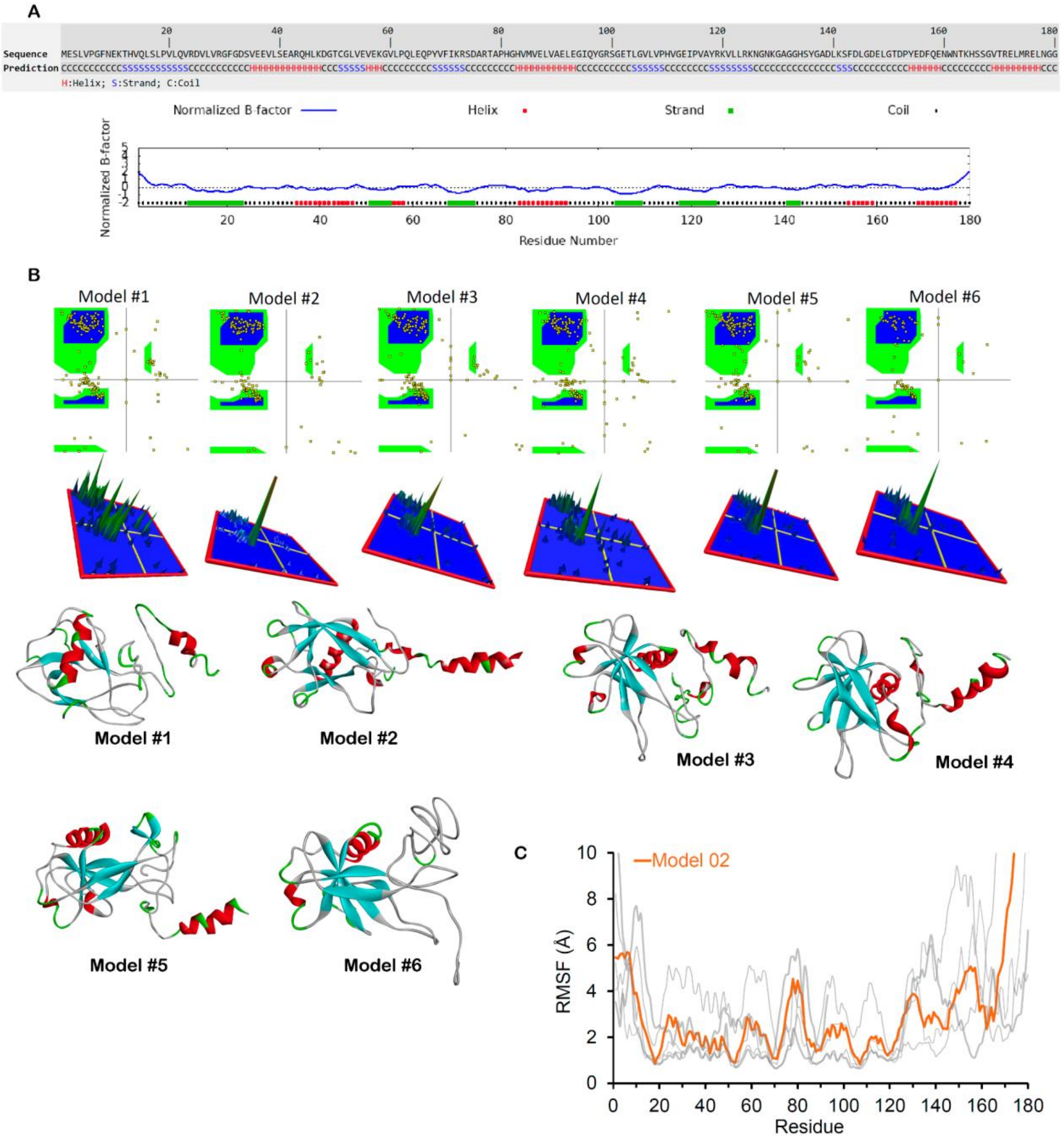

Figure S2. **(A)** The SARS-CoV-2 Nsp1 sequence compared with the predicted secondary structures using I-TASSER. The bottom panel represents the B-factor of individual model residues. **(B)** The Ramachandran plots in 2D and 3D secondary structure (α-helices and β-sheets) representation for other simulated models of Nsp1. The bottom panel represents the model structures of SARS-CoV-2 Nsp1. **(C)** RMSFs of simulated Nsp1 models during molecular dynamics simulations. In addition, model #2 is highlighted in orange color.

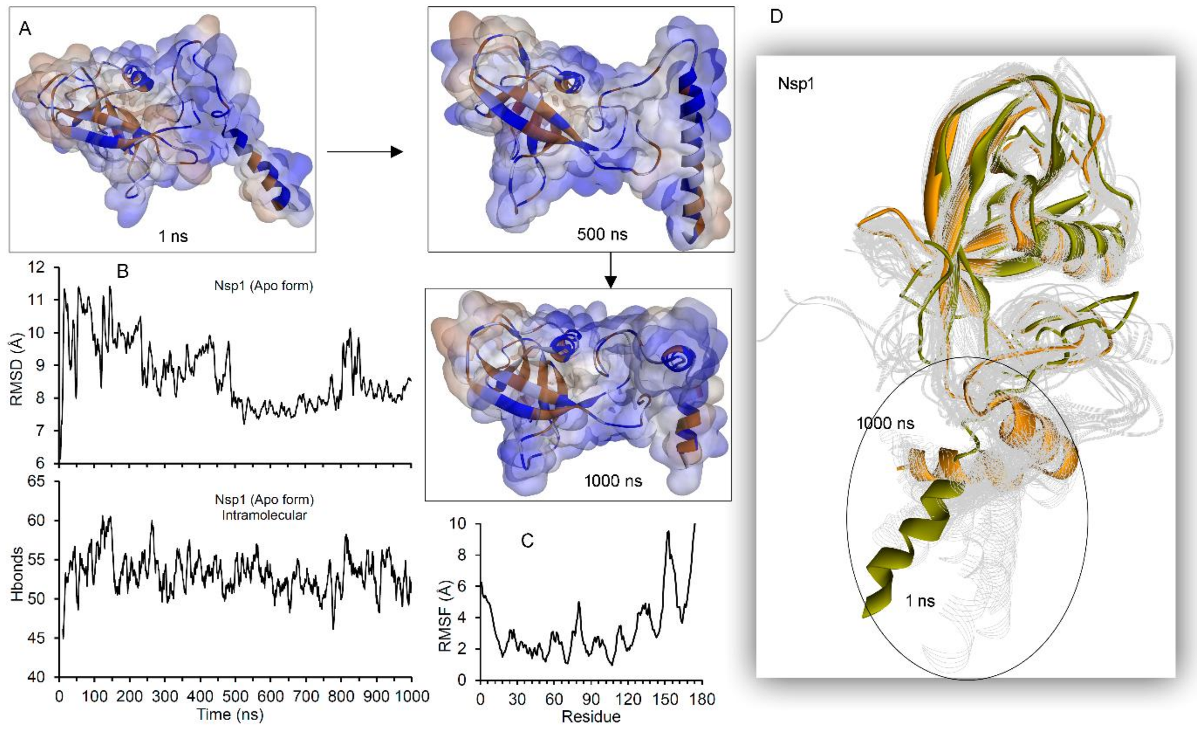

Figure S3. Conformational switch of the Nsp1 protein structure. **(A)** The changing surface electrostatics over time for the Nsp1 protein; hydrophobicity in blue for hydrophilic region and brown for hydrophobic region. **(B)** The RMSDs (root-mean-square deviation) from 1000 ns MD simulation, and the bottom plot represents hydrogen binding interaction (intramolecular) within the Nsp1 protein. **(C)** The root mean square fluctuation (RMSF data for simulated Nsp1 models. **(D)** Conformational switch of the Nsp1 C-terminal region, represented from the beginning and end of MD simulation time, along with protein coordinates from different time intervals (in gray).

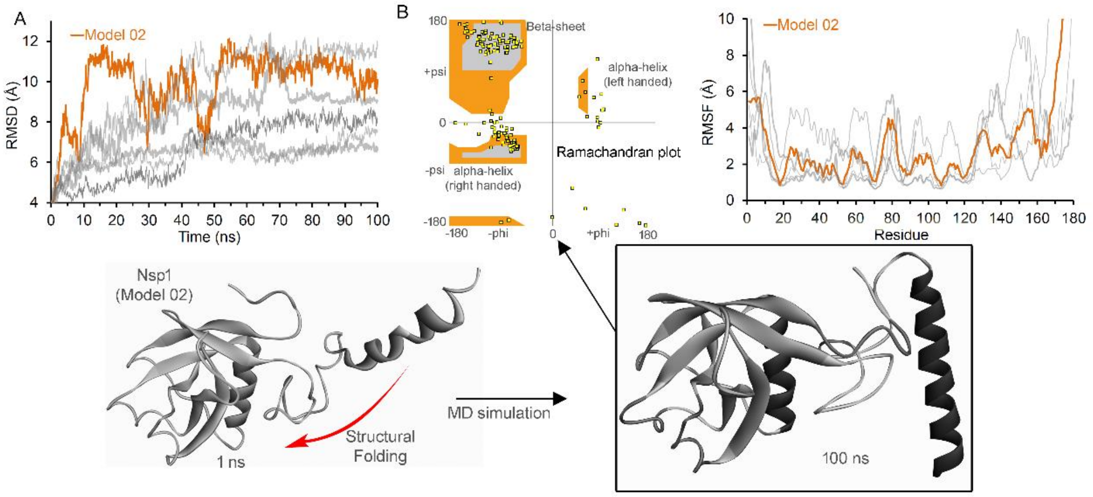

Figure S4. Protein-protein interactions of Nsp1 with cyclophilins or 40S ribosomal or RNA exosome proteins. The RMSD (left plot) and RMSF (right plot) data for all Nsp1 models during 100 ns MDS (for model 2 protein coordinates were extracted from 1000 ns). The central panel represents a well-defined secondary structure based on the Ramachandran plot (averaged Nsp1 model 2). The below protein structures represent conformational changes for the Nsp1 model 2, and the optimized structure after 100 ns.

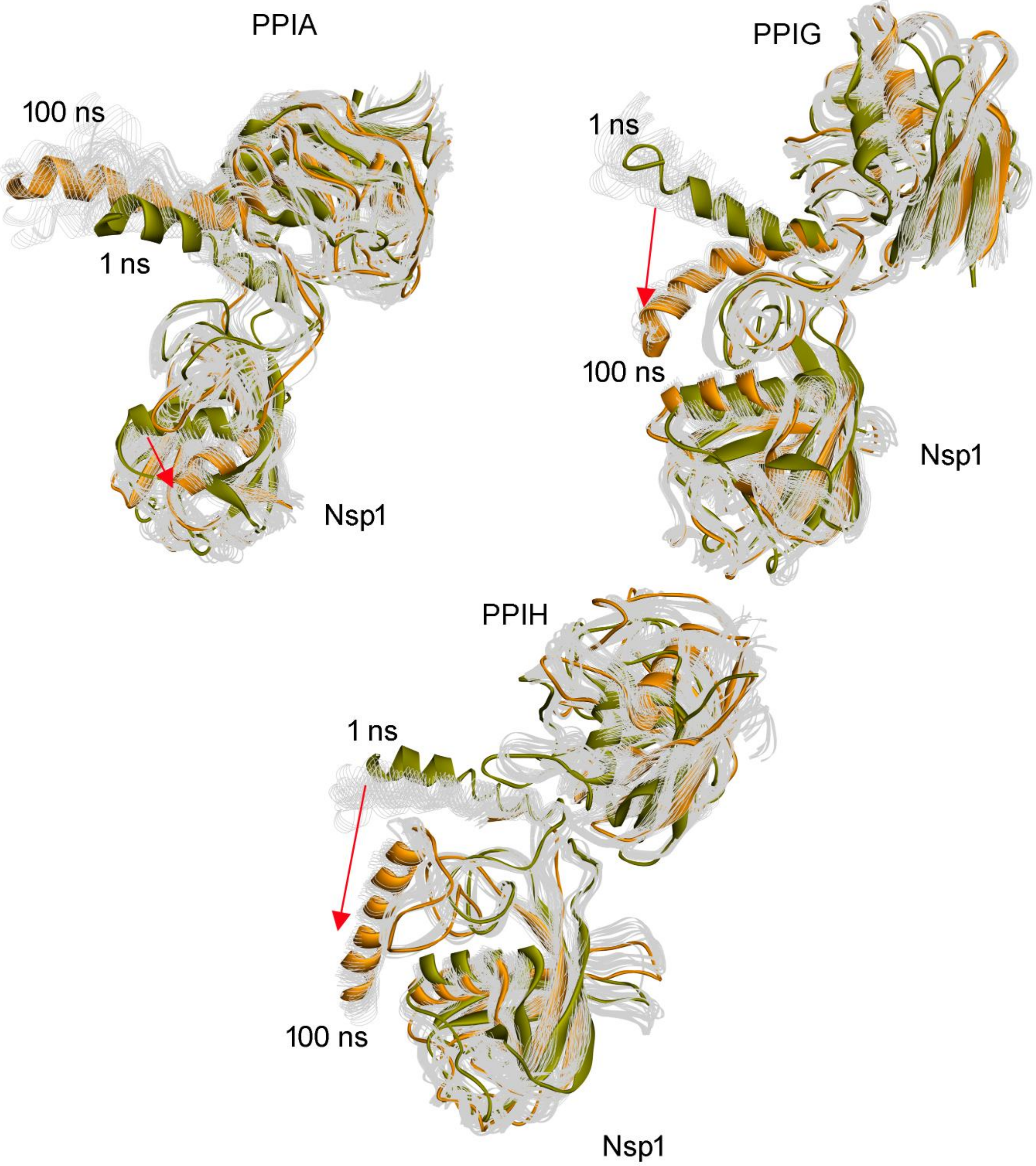

Figure S5. Distinct behavior of Nsp1 with respect to PPIA or PPIG or PPIH cyclophilins. Protein coordinates were extracted from different time frames of the MD simulation time.

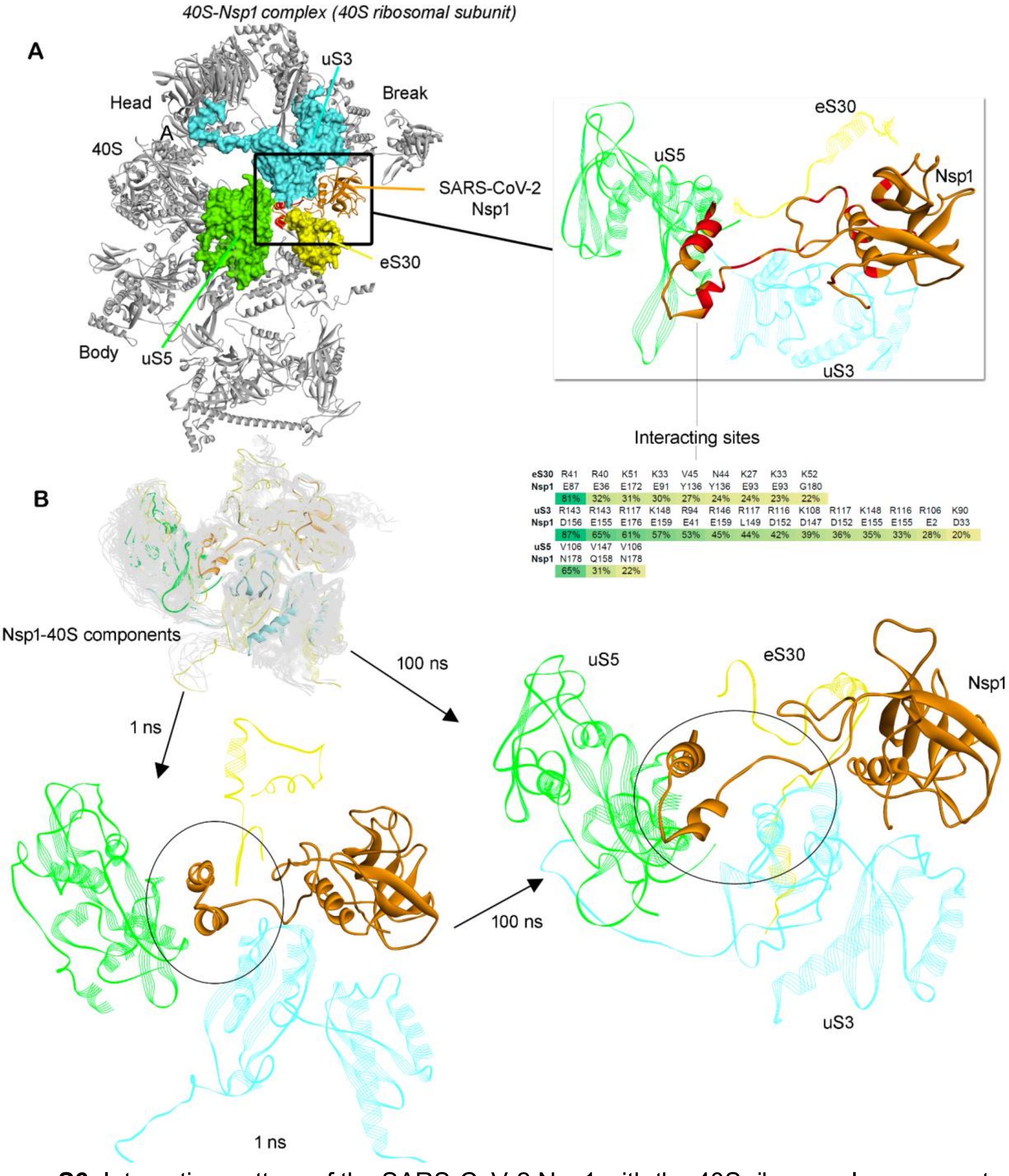

**eS30**	R41	R40	K51	K33	V45	N44	K27	K33	K52					
**Nsp1**	E87	E36	E172	E91	Y136	Y136	E93	E93	G180					
	81%	32%	31%	30%	27%	24%	24%	23%	22%					
**uS3**	R143	R143	R117	K148	R94	R146	R117	R116	K108	R117	K148	R116	R106	K90
**Nsp1**	D156	E155	E176	E159	E41	E159	L149	D152	D147	D152	E155	E155	E2	D33
	87%	65%	61%	57%	53%	45%	44%	42%	39%	36%	35%	33%	28%	20%
**uS5**	V106	V147	V106											
**Nsp1**	N178	Q158	N178											
	65%	31%	22%											

Figure S6. Interaction pattern of the SARS-CoV-2 Nsp1 with the 40S ribosomal components. **(A)** Nsp1 conformation with the 40S ribosomal subunit, studied applying our molecular dynamic simulations (pdb id.: 6zoj). For the Nsp1-40S complex, our energy minimized Nsp1 full-length (after 100 ns of MD simulation) structure (in orange) was replaced with the fragmented C-terminus structure in the 40S ribosome cryo-EM structure (pdb id.: 6zoj; in red). The right panel represents hotspot binding sites from the Nsp1 protein with the 40S components (uS5, uS3, and eS30), residing in the C-terminal region. The below panel describes hydrogen bonding residues from individual components, and long-lasting interactions (with occupancy > 20%). **(B)** Conformational changes of the Nsp1-40S components observed during 100 ns MD simulation time.

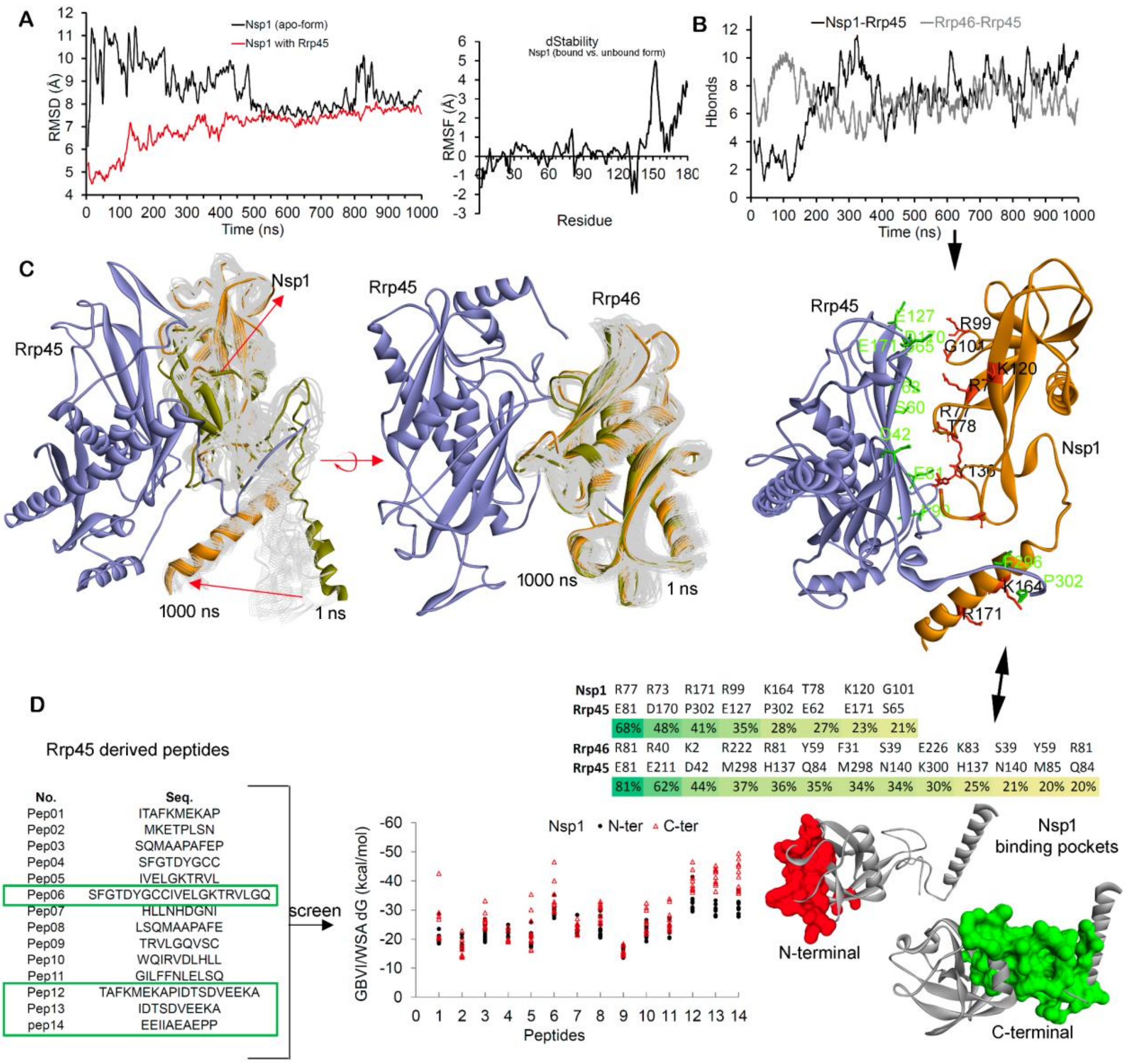

Figure S7. Investigating the hypothesized Nsp1-Rrp45 complex and comparing it with the Rrp45-Rrp46 interactions from RNA exosomes. The eukaryotic RNA exosome consists of six RNase proteins (Rrp41, Rrp42, Rrp43, Rrp45, Rep46, and Mtr3; pdb id.: 2nn6) and three RNA binding proteins. Nsp1 / Rrp46 were investigated to trace binding efficiency with the Rrp45 from the exosome complex. **(A)** The RMSDs of Nsp1 in bound and unbound state with Rrp45. The right panel represents change in stability (RMSFs) of Nsp1 when bound with Rrp45, compared with the apo-form or unbound state. **(B)** Protein-protein intermolecular hydrogen bond interactions of Nsp1-Rrp45 and Rrp46-Rrp45 (pdb id.: 2nn6) complexes. Below panel represents high occupancy intermolecular interactions (≥ 20 %) between the Nsp1-Rrp45 and Rrp45-Rrp46 components. The right panel represents interactions from the Nsp1-Rrp45 system. **(C)** The dynamics of Nsp1 or Rrp46 when complexed with the Rrpp45 protein, individual protein coordinates were extracted from different time frames of the MD simulations. **(D)** Rrp45 peptide candidates retrieved from PPI, which could be considered to block the activity of the SARS-CoV-2 Nsp1 protein. In addition, these peptides were further investigated using the MD simulations approach. The right panel represents two active sites predicted using the Alpha Shape approach in the MOE package (Molecular Operating Environment; Chemical Computing Group Inc.) over Nsp1 and considered for peptide screens.

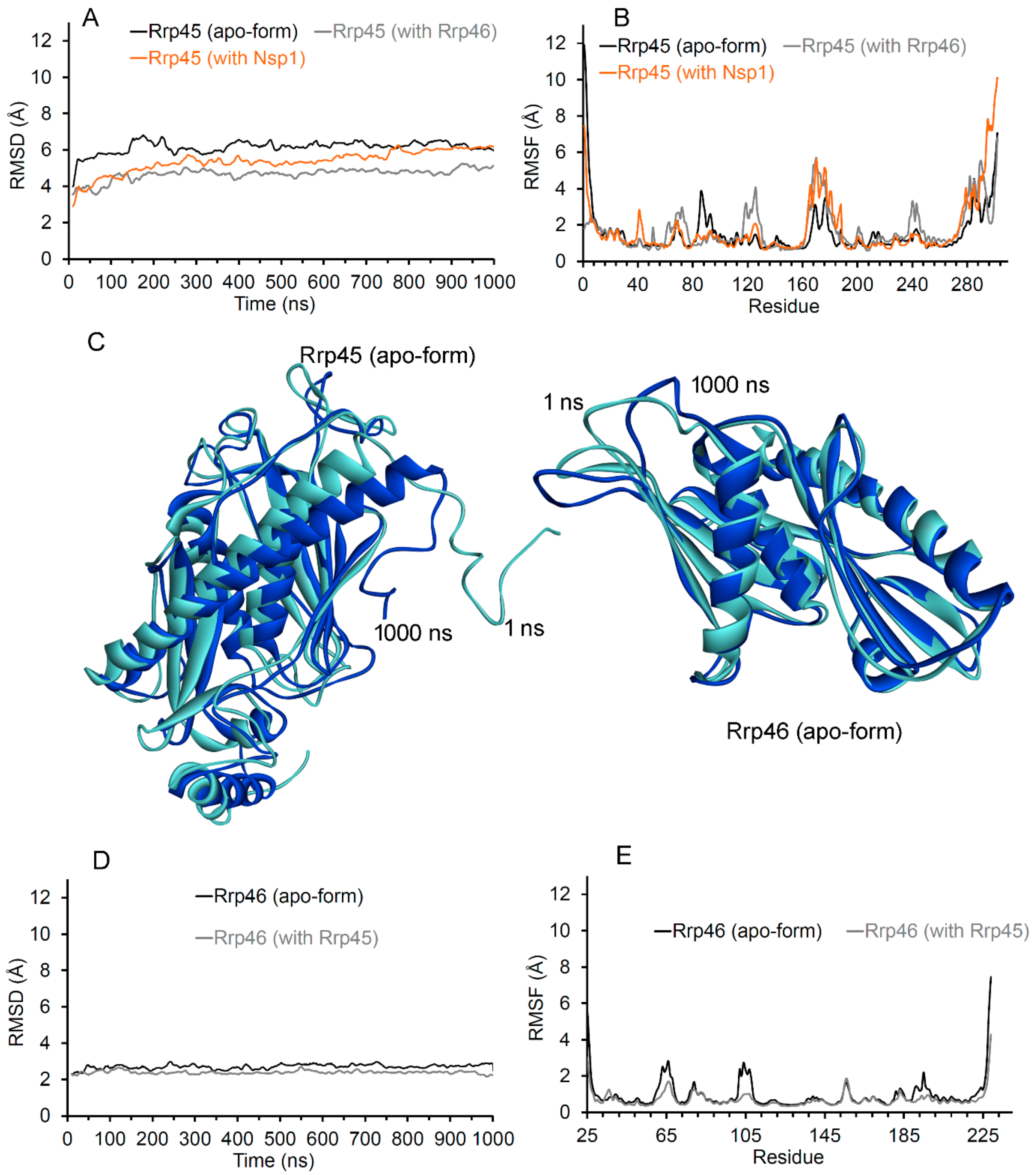

Figure S8. Rrp45 and Rrp46 structural properties. **(A)** and **(B)** RMSD and RMSFs of Rrp45 in the presence or absence of Rrp45, respectively. The orange line represents Rrp45 from the Nsp1-Rrp45 system. **(C)** Conformational dynamics of the apo-form of Rrp45 or Rrp46 during 1000 ns of MD simulation time. **(D)** and **(E)** RMSDs and RMSFs of Rrp46 in the presence or absence of Rrp45, respectively.

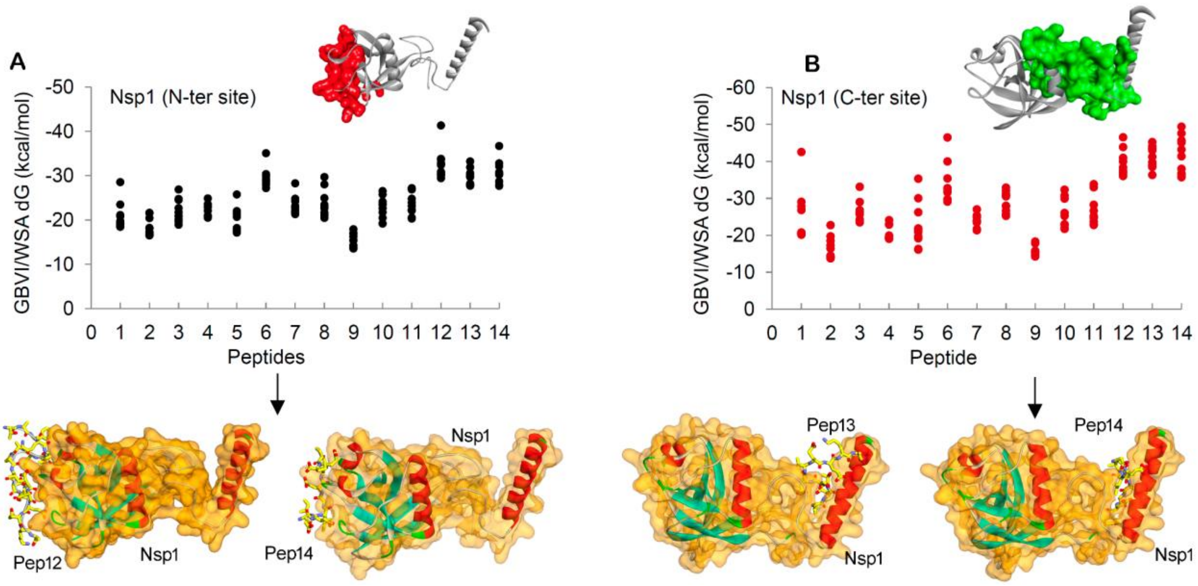

Figure S9. Screening of the Rrp45 derived peptides against two distinct predicted active sites for SARS-CoV-2 Nsp1. **(A)** and **(B)** Binding affinity (GBVI/WSA dG; kcal/mol) with the N-terminus and C-terminus sites from Nsp1 of the Rrp45 derived peptides. The center table represents a list of peptides, and the box marked with green are peptides showing high affinity with both predicted sites in Nsp1. Green backgrounds are peptides with high affinity on N-ter active sites and gray backgrounds only on C-ter active sites. The best binding two peptides (below panel) with the Nsp1 protein were further investigated using the MD simulation approach. The binding affinity (GBVI/WSA dG; kcal/mol) of each peptide with Nsp1 was retrieved from the MOE pipeline (Molecular Operating Environment; Chemical Computing Group Inc.).

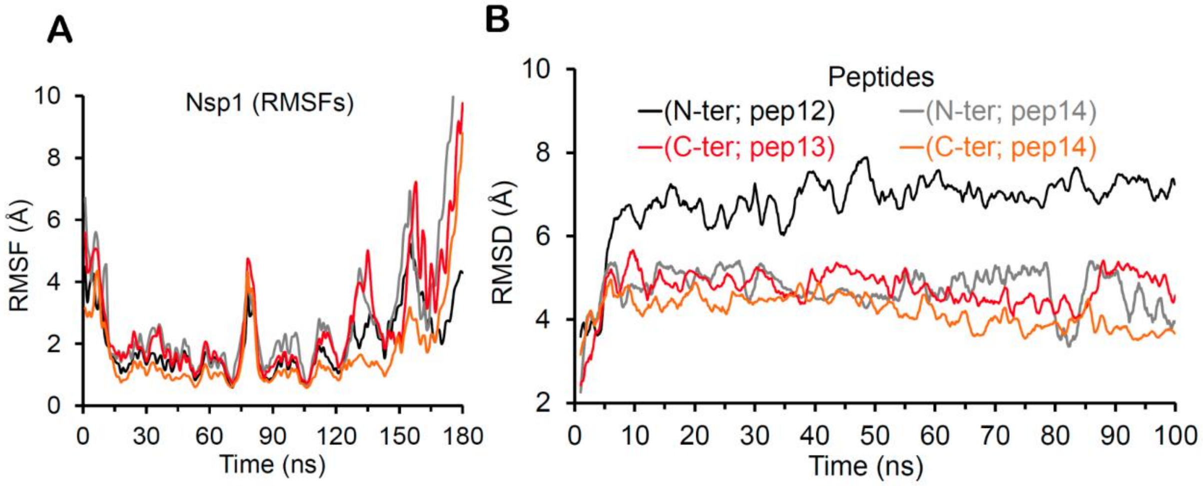

Figure S10. Screening of the Rrp45 derived peptides against two distinct predicted active sites for SARS-CoV-2 Nsp1. **(A)** Stability of the Nsp1 protein with different peptides (pep12; TAFKMEKAPIDTSDVEEKA, pep13; IDTSDVEEKA, and pep14; EEIIAEAEPP) represented as RMSDs and RMSFs. **(B)** The RMSDs of individual peptides when complexed with the SARS-CoV-2 Nsp1 protein.

Table S2. High occupancy interacting residues from cyclophilins (PPIA, PPIG, PPIH, FKBP1A, and FKBP1B) and C-termini residues of the SARS-CoV-2 Nsp1. Individual interactions with occupancy ≥ 10 % are presented in this table.

FKBP1A	Nsp1		FKBP1B	Nsp1		PPIA	Nsp1		PPIG	Nsp1		PPIH	Nsp1	
Y82	N160	32.83%	K57	E155	76.35%	R55	E159	40.52%	R7	D156	59.58%	D54	W161	17.37%
G86	K164	31.54%	F59	E155	71.66%	K125	D156	25.15%	Q143	D156	27.05%			
T85	K164	12.87%	G58	E155	71.66%	K125	E155	24.55%	K103	Q158	26.35%			
Y82	K164	11.48%	Y82	E155	32.83%	T119	W161	22.75%	N105	H165	25.05%			
			K44	E148	31.84%				R9	E155	24.35%			
			R49	E41	29.64%				N105	Q158	20.26%			
			K52	D144	25.85%				N105	N162	16.57%			
			R49	D48	23.75%				A107	W161	15.87%			
			D37	K164	22.95%				Q143	F157	12.87%			
			E54	K47	20.76%									
			D147	K47	20.26%									
			D152	K52	19.66%									
			I56	E155	14.47%									
			K47	Y154	12.97%									
			K52	D48	12.67%									
			D37	N160	12.38%									

Table S3. Intermolecular hydrogen bond interactions between the SARS-CoV-2 Nsp1 and derived peptides from the Rrp45 protein. Long lasting interactions with occupancy ≥ 1.00% are presented in this table.

**N-terminal site**						**C-terminal site**					
**Nsp1-pep12**			**Nsp1-pep14**			**Nsp1-pep13**			**Nsp1-pep14**		
**Pep12**	**Nsp1**		**Pep14**	**Nsp1**		**Pep13**	**Nsp1**		**Pep14**	**Nsp1**	
E6	K58	58.68%	E1	R73	65.07%	E8	R171	43.61%	E1	S40	55.69%
K7	K58	32.83%	E2	R119	41.52%	K9	D147	9.68%	E1	E37	33.63%
D14	R24	30.64%	P10	R99	19.76%	A10	K141	9.48%	E2	R171	22.55%
E6	R99	27.35%	P10	K58	14.97%	E7	R171	5.69%	P9	K129	20.86%
E6	R99	20.56%	E6	K58	6.59%	S4	E37	4.59%	I4	G132	17.47%
A19	R24	19.96%	P9	K58	4.79%	I1	E36	3.69%	E8	K129	13.57%
E16	R24	17.56%	I4	K58	3.29%	D2	S40	3.49%	I3	L145	9.98%
K18	E57	10.08%	E1	K120	2.50%	K9	E37	3.19%	E8	A131	9.58%
D11	K58	9.08%	E8	P80	2.00%	V6	N178	2.79%	P10	K129	3.99%
K4	R99	8.08%				E8	M174	2.10%	E2	L145	2.59%
E17	R24	7.49%							E1	R43	2.30%
K4	E93	7.09%									
E17	K58	2.00%									

Video S1 (*attached as a separate file*). Conformational dynamics of the Nsp1 protein (apo-form) throughout 1000 ns MD simulation, representing the structural folding in C-terminal region.
